# Adolescent Hippocampal and Prefrontal Brain Activation During Performance of the Virtual Morris Water Task

**DOI:** 10.3389/fnhum.2018.00238

**Published:** 2018-06-26

**Authors:** Jennifer T. Sneider, Julia E. Cohen-Gilbert, Derek A. Hamilton, Elena R. Stein, Noa Golan, Emily N. Oot, Anna M. Seraikas, Michael L. Rohan, Sion K. Harris, Lisa D. Nickerson, Marisa M. Silveri

**Affiliations:** ^1^Neurodevelopmental Laboratory on Addictions and Mental Health, McLean Hospital, Belmont, MA, United States; ^2^Department of Psychiatry, Harvard Medical School, Harvard University, Boston, MA, United States; ^3^Department of Psychology, University of New Mexico, Albuquerque, NM, United States; ^4^School of Medicine, Boston University, Boston, MA, United States; ^5^Brain Imaging Center, McLean Hospital, Belmont, MA, United States; ^6^Boston Children’s Hospital, Harvard Medical School, Harvard University, Boston, MA, United States; ^7^Applied Neuroimaging Statistics Laboratory, McLean Hospital, Belmont, MA, United States

**Keywords:** adolescence, BOLD fMRI, Morris water task, spatial memory, hippocampus, prefrontal cortex

## Abstract

The frontal cortex undergoes substantial structural and functional changes during adolescence and significant developmental changes also occur in the hippocampus. Both of these regions are notably vulnerable to alcohol and other substance use, which is typically initiated during adolescence. Identifying measures of brain function during adolescence, particularly before initiation of drug or alcohol use, is critical to understanding how such behaviors may affect brain development, especially in these vulnerable brain regions. While there is a substantial developmental literature on adolescent working memory, less is known about spatial memory. Thus, a virtual Morris water task (vMWT) was applied to probe function of the adolescent hippocampus. Multiband blood oxygen level dependent (BOLD) functional magnetic resonance imaging (fMRI) data were acquired at 3T during task performance. Participants included 32 healthy, alcohol- and drug-naïve adolescents, 13–14 years old, examined at baseline of a 3-year longitudinal MRI study. Significantly greater BOLD activation was observed in the hippocampus and surrounding areas, and in prefrontal regions involved in executive function, during retrieval relative to motor performance. In contrast, significantly greater BOLD activation was observed in components of the default mode network, including frontal medial cortex, during the motor condition (when task demands were minimal) relative to the retrieval condition. Worse performance (longer path length) during retrieval was associated with greater activation of angular gyrus/supramarginal gyrus, whereas worse performance (longer path length/latency) during motor control was associated with less activation of frontal pole. Furthermore, while latency (time to complete task) was greater in females than in males, there were no sex differences in path length (accuracy), suggesting that females required more time to navigate the virtual environment, but did so as effectively as males. These findings demonstrate that performance of the vMWT elicits hippocampal and prefrontal activation patterns in early adolescence, similar to activation observed during spatial memory retrieval in adults. Given that this task is sensitive to hippocampal function, and that the adolescent hippocampus is notably vulnerable to the effects of alcohol and other substances, data acquired using this task during healthy adolescent development may provide a framework for understanding neurobiological impact of later initiation of use.

## Introduction

While the frontal cortex undergoes substantial and rapid structural and functional changes during adolescence, significant developmental changes also occur in the medial temporal lobe, specifically the hippocampus, which is responsible for learning and memory (Spear, 2000; Paus, 2005; Mills et al., 2014). Neurodevelopmentally, dynamic integration of hippocampal and prefrontal circuitry is necessary to incorporate experience into behaviors that are ultimately adaptive for successful developmental transitions (Murty et al., 2016). These regions also are notably susceptible to alcohol, cannabis, and stimulant use (Conrad et al., 2016), the onset of which typically overlaps with this crucial period of adolescent brain development. Thus, identifying neurobiological precursors of use and vulnerabilities associated with early and escalating substance use during adolescence is critical (Casey and Jones, 2010).

The hippocampus is involved in the spatial layout and structural representation of an environment in both rodents (O’Keefe and Nadel, 1978; Sutherland et al., 1989; Jarrard, 1993) and humans (Maguire et al., 1999, 2000) and also serves spatial memory processing (O’Keefe and Nadel, 1978; Guderian et al., 2015). The Morris water task (MWT) (Morris, 1984) has been used extensively in animal research to probe spatial memory ability and related hippocampal circuitry. Virtual MWT (vMWT) versions also have been developed to assess spatial memory ability in humans (Hamilton et al., 2009), providing an important translational approach for bridging current knowledge of memory function across species. Several studies have confirmed that the hippocampus is essential for solving a spatial navigation challenge, e.g., using cues in an environment to successfully and efficiently navigate to a hidden platform (Astur et al., 2002, 2004; Bohbot et al., 2004; Hamilton et al., 2009; Sneider et al., 2015). Spatial memory paradigms have been paired with blood oxygen level dependent (BOLD) functional magnetic resonance imaging (fMRI) to characterize neurocircuitry involved in task performance (Sneider et al., 2011, 2013; Dahmani and Bohbot, 2015; Woolley et al., 2015; Pu et al., 2017). On a version of the vMWT similar to the one used in the current study, there were no significant sex differences on behavioral measures, however, adult females exhibited greater left lateralized hippocampal activity compared to adult males that was specific to fMRI learning trials (not retrieval) (Sneider et al., 2011). In a study of chronic marijuana (MJ) users, the MJ group showed equivalent learning to that observed in non-users, yet demonstrated a deficit in memory retrieval performance that was accompanied by hypoactivation of right parahippocampal gyrus and cingulate gyrus (Sneider et al., 2013). There are only two other studies published to date that have employed a similar vMWT (all in adult cohorts), one that was paired with magnetoencephalography (MEG) and the other that was paired with resting state fMRI. In the MEG study, greater left hippocampal and parahippocampal theta activity was reported in healthy male adults during directional navigation relative to random navigating, findings interpreted as reflecting hippocampal place cell activity implicated in memory formation (Pu et al., 2017). In the resting state fMRI functional connectivity study, adult participants performing the vMWT demonstrated increased functional connectivity between left posterior hippocampus and left dorsal caudate that was specific to the learning session (i.e., pre- versus post-learning), with the magnitude of the increase being correlated with offline gains in performance (Woolley et al., 2015). Results of these vMWT studies are consistent with another type of spatial task used during fMRI – a virtual concurrent spatial discrimination learning task (12-arm radial maze) – in which BOLD activation was observed in hippocampus, prefrontal cortex, and caudate nucleus in healthy 18- to 35-year olds during navigation performance (Dahmani and Bohbot, 2015). Taken together, these studies underscore the involvement of hippocampus, and in some cases the coupling of hippocampus with executive function neurocircuitry, during spatial memory task performance across neuroimaging modalities and different types of tasks.

Not surprisingly, spatial memory ability and behavioral performance on the vMWT decline in the elderly (Etchamendy et al., 2012), and are impaired in individuals with hippocampal damage (Astur et al., 2002). While there have been substantial studies documenting normative development of working memory during adolescence (e.g., Squeglia et al., 2013; Andre et al., 2016; Montez et al., 2017), the literature on this type of vMWT spatial memory during adolescent development does not yet exist. To this end, the objective of the current study was to use the vMWT with BOLD fMRI to investigate hippocampal and executive function neurocircuitry during task performance in 13- to 14-year-old healthy adolescents. These results represent baseline data from an ongoing longitudinal study of adolescent brain development, for which participants were alcohol- and drug-naïve and who had no psychiatric diagnoses. Hippocampal and prefrontal activation have been observed during memory retrieval on this task in adults, therefore, it was hypothesized that significant hippocampal and prefrontal activation also would be observed in healthy adolescents. Furthermore, given that differences between males and females in spatial memory performance, on this task and others, have been well established (Astur et al., 2004; Newhouse et al., 2007; Andreano and Cahill, 2009; Woolley et al., 2010; Sneider et al., 2015; Voyer et al., 2017; Piber et al., 2018), sex differences in performance and BOLD fMRI data were examined. Examination of brain activation during performance of an established translational vMWT provides an important opportunity to characterize normative hippocampal and prefrontal contributions to memory retrieval in physically and psychologically healthy adolescents who were substance-naïve. Given that integration of these regions is important for enhancing decision-making skills during a crucial decade of brain development (Murty et al., 2016), and that alterations in hippocampal and prefrontal functioning have been implicated in substance use and other psychiatric disorders (e.g., Chambers et al., 2003; Bava and Tapert, 2010; Lichenstein et al., 2016; Silveri et al., 2016), data from this study establish an important baseline that may help elucidate neurobiological markers of risk for an early initiation of substance use, as well as manifestation of psychiatric symptoms (e.g., depression and anxiety) that tend to emerge during adolescence.

## Materials and Methods

### Participants

The study sample consisted of 32 healthy adolescents (15 females) who completed the baseline visit of a 3-year longitudinal study of adolescent brain development. Demographic details are provided in **Table 1**. The clinical research protocol was approved by the Partners Healthcare Institutional Review Board of McLean Hospital. Participants were recruited through Boston Children Hospital’s (BCH) Research Participant Registry (which involves recruitment of adolescent participants across local pediatrician clinics) and local advertisements. Interested participants were subsequently screened via an online eligibility survey and completed follow-up verification and scheduling via telephone. All participants and their parent(s) or guardian(s) provided written informed assent and consent, respectively, after they received a complete description of the study. Monetary compensation was provided to all participants for study completion.

**Table 1:** Participant demographics and cognitive measures.

	Total sample (*n* = 32)
Age (years)	13.9 ± 0.7
Female/Male	15 (47%)/17 (53%)
Education (years)	7.7 ± 0.9
SES^a^	49.4 ± 9.4
Handedness	30R, 2L
Ethnicity^b^	97% Non-Hispanic
Race^c^	75% Caucasian
	12% Asian
	13% Other
WASI T-Scores	
IQ estimate (2-subtest, vocabulary/matrix)	114.3 ± 9.6
Vocabulary	59.4 ± 7.5
Matrix reasoning	56.8 ± 6.5
Block design	57.0 ± 9.0


*Data represent means ± standard deviations. SES, socioeconomic status; WASI, Wechsler Abbreviated Scale of Intelligence. ^*a*^SES (Hollingshead, 1975); ^*b*^Ethnicity: Hispanic vs. Non-Hispanic; ^*c*^“Other” included the following designations: Asian/Caucasian; African American/Caucasian; American Indian or Native Alaskan/Caucasian. There were no significant sex differences on any measure.*

Participants completed urine screening prior to scanning to rule out current psychoactive substance use (Clarity Diagnostics Drugs of Abuse Panel, Boca Raton, FL, United States) and pregnancy (QuPID One-Step Pregnancy, Stanbio Laboratory, Inc., San Antonio, TX, United States). Participants had no prior head trauma with loss of consciousness, were free of radiologic brain abnormalities and MR scanning contraindications, and had no lifetime psychoactive substance use, including nicotine or alcohol, and had no previous or current diagnosis of any psychiatric condition.

### Clinical and Cognitive Measures

Trained staff conducted diagnostic clinical interviews using the Mini International Neuropsychiatric Interview for Children and Adolescents (MINI-KID) (Sheehan et al., 2010). The vocabulary and matrix reasoning subtests of the Wechsler Abbreviated Scale of Intelligence (Wechsler, 1999) were administered to obtain an estimate of general intelligence, and the block design subtest was administered to assess visuospatial processing (**Table 1**).

### Virtual Morris Water Task (vMWT)

Participants completed offline vMWT training prior to performing the task in the MRI scanner. For additional details of the virtual vMWT, see the prior publications (Hamilton et al., 2009; Sneider et al., 2013, 2015). A laptop using a Windows operating system was used to administer vMWT both offline and during fMRI. The laptop displayed a first-person perspective of a virtual environment: a circular pool within a square room. A square platform was either hidden or visible according to trial design and was located in the southwest (SW) quadrant of the virtual pool environment for all trials.

**FIGURE 1:**
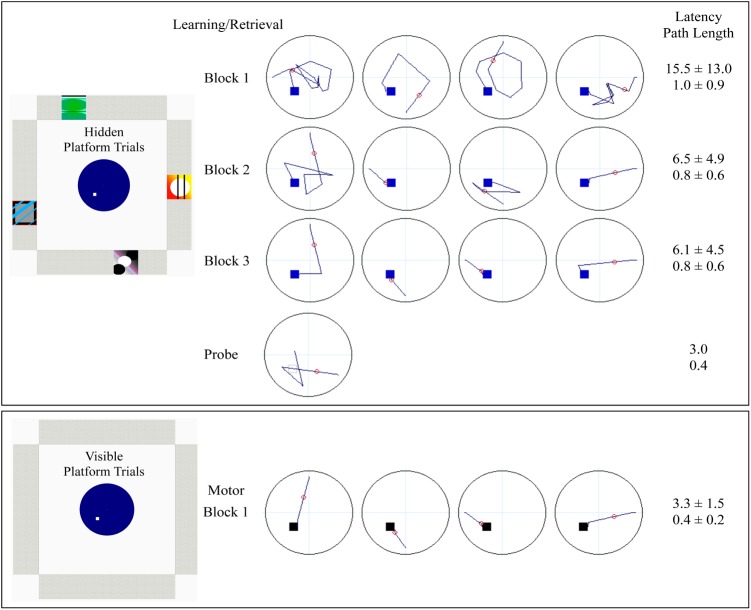
Schematic denoting the layout of offline vMWT training for learning/retrieval, probe and motor control. Learning/retrieval consisted of three blocks of four hidden platform trials, each starting from a unique direction (in pseudorandom order). The probe was a single trial, with a start location of either NW or the SE (pseudorandom), which were equidistant from the platform location in the SW. Dark blue lines represent sample paths from a single study participant completed each condition. The small squares within the pool represent platform location (blue: hidden; gray open: probe; black: visible). The small red circle is used to calculate heading error (only calculated for the probe). Accompanying latency (seconds) and path lengths, averaged over block for sets of four trials, or for the single probe trial, are provided from the study participant. Decreased latency (speed of performance) and decreased path length (accuracy independent of speed) indicate successful learning.

Offline training consisted of learning/retrieval, probe and motor conditions (**Figure 1**). Participants first completed 12 learning/retrieval trials, where the platform was hidden below the surface of the water in a virtual environment featuring distinct spatial cues (pictures) on each of the four walls. Participants completed three blocks of four trials, in which each of the four trials began in a unique location, north, south, east or west (pseudorandom order). Participants had up to 60 s per trial to navigate to the hidden platform, at which point a “platform found” message was presented on the screen. If participants did not find the platform (unsuccessful), the platform becomes visible until they successfully navigate to it. The inter-trial interval was 1 s. The next trial was a single probe trial, in which the environment was the same as the prior 12 learning/retrieval trials, however, the platform was removed unbeknownst to participants, who search for 30 s before the trial ends. Participants then completed one block of four motor trials, which served as a motor performance control condition, where the platform was visible above the surface of the water in the same virtual environment but with no spatial cues on any of the four walls. Participants also had up to 60 s to complete the motor trials. Participants navigated through the virtual environment using laptop keyboard arrow keys (right, left, and forward) for offline training. For the learning/retrieval, probe and motor conditions, the virtual environments, release points, platform location, layout of blocks and trials, and sample paths, latency and path lengths for one study participant are illustrated in **Figure 1**.

**FIGURE 2:**
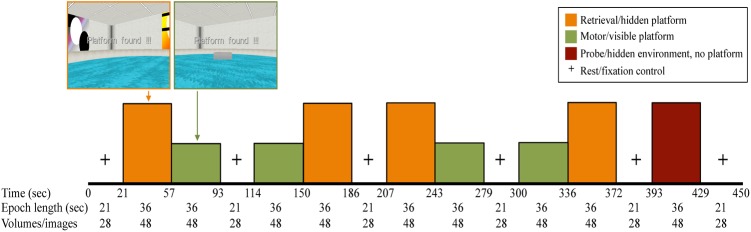
fMRI paradigm in which all participants completed four retrieval (orange) and four motor conditions (green) (36-s epochs), interspersed with rest periods where participants viewed a fixation cross on the screen (21 s). The last performance block was a 36-s probe trial (maroon), in which the platform was removed from the pool unbeknownst to the participant.

The fMRI vMWT paradigm (**Figure 2**) utilized a block design, consisting of pairs of alternating retrieval (left/orange) and motor (right/green) blocks (four of each, 36 s per block) (Shipman and Astur, 2008) separated by rest blocks (fixation cross, 21 s per block), and ending with a probe trial (36-s block). For retrieval and motor trials, participants completed as many trials as possible with fixed 36-s blocks. For the probe trial, in which the cues in the virtual environment were the same as in the retrieval trial only with the platform removed, participants navigated for the entire duration of the 36-s block. Navigation was controlled using an MRI-compatible fiber optic response pad (fORP) (diamond configuration) during BOLD fMRI acquisition.

For offline training and fMRI, vMWT performance measures included latency to reach the platform (time from first movement to reaching the platform, measured in seconds) and path length (distance traveled from release point to platform/end of trial, measured in arbitrary units). Significant decreases in latency and path lengths over trial blocks were interpreted as successful learning of the platform location. Sum of completed trials for retrieval and motor conditions during fMRI also were determined, indicating that all participants experienced successful navigation on both trial types. Data from one female subject was not available for offline training due to computer error during data acquisition.

**FIGURE 3:**
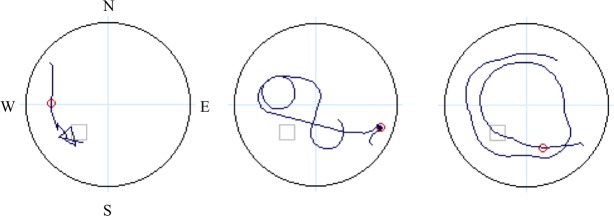
Schematic denoting four quadrants (light blue lines: N, E, S, W), with dark blue lines representing sample paths from three study participants during navigation on the probe trial. The small gray open square is the location of the hidden platform and the small red circle is used to calculate heading error.

For the offline probe trial, eight dependent measures were calculated: first move latency, latency to critical region, path length to critical region, % path length in critical region, latency to quadrant, path length to quadrant, % path length in quadrant, and heading error. “Quadrant” refers to the SW quadrant of the virtual space, which contains the platform (**Figures 1**, **3**), while “critical region” refers to a more circumscribed circular region (radius ∼12% of the pool diameter) centered around the platform. “Heading error” refers to the angular deviation of the participant’s path from a direct path from the release point to the platform, calculated at the point where the cumulative path length first exceeds an amount equal to 25% of the pool diameter.

Independent raters blind to participant age and sex determined the navigation strategy utilized during performance of the offline probe trial: (1) a direct strategy, where participants navigated directly to the platform location from the starting location; or (2) a non-direct strategy, where participants navigated in a circuitous or random route that was not in the direction of the platform quadrant (NE) (Astur et al., 2004). Sample navigation paths are provided in **Figure 3**. Two coders (JS and MS), who independently viewed individual output files of navigation maps produced during the offline probe trial classify strategy, had complete agreement between rates, with an intraclass correlation coefficient for strategy coding of ICC = 1.00, *p* < 0.001. Given the shortened testing time of the probe trial during fMRI, and therefore a lack of comparability with offline probe performance, dependent measures from the fMRI probe trial are not presented in this report.

### Functional Magnetic Resonance Imaging Acquisition and Preprocessing

Functional data were acquired on a Siemens TIM Trio 3 Tesla MRI system (Erlangen, Germany) with a 32-channel head coil. High-resolution structural images were collected using a T1-weighted multi-echo magnetization prepared rapid acquisition gradient echo (ME-MPRAGE) 3D sequence in four echoes (TE = 1.64/3.5/5.36/7.22 ms, TR = 2.1 s, TI = 1.1 s, FA = 12°, 176 slices, 1 mm × 1 mm × 1.3 mm voxel, acquisition time = 5 min) for registration of functional images into standard space. Whole-brain multiband gradient echo echo-planar imaging (EPI) with BOLD contrast was used to collect fMRI data in one 7.5 min run. Images were acquired in 54 interleaved oblique slices (TR/TE/FA = 750 ms/30 ms/52°, FOV = 220, voxel size: 2.8 mm × 2.8 mm × 2.8 mm, multiband = 6, GRAPPA = 2). A fieldmap was acquired at the same resolution and slice locations to allow for B0 unwarping (TR = 1000, TE = 10/12.46 ms, FA = 90°, 2:44 min).

Prior to statistical analyses, preprocessing was performed on raw functional images using the FMRIB Software Library (FSL) software v5.0.10 (Smith et al., 2004) (FMRIB, Oxford, United Kingdom), (Groves et al., 2009) including: motion correction, slice-timing correction, non-brain removal, spatial smoothing (FWHM 6 mm Gaussian kernel), and grand-mean intensity normalization of the 4D dataset by a single multiplicative factor. Ten volumes at the onset of the first rest block were removed to allow for signal equilibration. While data were initially acquired from 40 healthy adolescent subjects, eight subjects were removed from the analysis due to excessive motion in the scanner. Any subject with greater than 5 mm motion was excluded from further analysis. For the remaining 32 subjects, ICA AROMA, an independent component analysis-based denoising tool, was then used to remove motion-related components from the fMRI data (Pruim et al., 2015^1^). While ICA-AROMA identifies components related to motion, components related to respiration and other artifacts also were identified by visual inspection of ICA components, then all motion-related and artifacts removed from the fMRI data using fsl_regfilt. Denoised fMRI data were then temporally filtered using a Gaussian-weighted least-squares straight line fit with a high-pass cutoff = 100 s and underwent fieldmap based distortion correction. Functional MRI data were registered to MNI152 standard space by first registering the fMRI images to the high-resolution structural image using boundary-based registration (BBR) and then transforming into MNI stereotaxic space using the first registration step combined with the registration information from registering the high-resolution structural image to MNI152 standard space, which was done using FNIRT.

### Statistical Analyses

Analysis of the vMWT performance measures (first movement latency, total latency, and total path length) were conducted using repeated measures analysis of variance (ANOVA) for trial block (three blocks for offline and four blocks for fMRI), with sex included as an independent variable. Number of completed trials also was analyzed over retrieval and motor trial blocks during fMRI using repeated measures ANOVAs, with sex included as an independent variable. *Post hoc* analyses for ANOVAs were conducted using two sample *t*-tests (two-tailed) to determine sources of differences when main effects or interactions were statistically significant. For the probe trial, percentage of overall path length was tested relative to chance for region (5%) and quadrant (25%) using one sample *t*-tests. Qualitative evaluations of navigation paths (spatial strategy employed, e.g., direct vs. non-direct) were quantitatively analyzed using chi-square non-parametric analyses. All statistical analyses for non-imaging measures were conducted using SPSS 24.0 (SPSS, Chicago, IL, United States). Cohen’s effect sizes (ES) were calculated for repeated measures ANOVAs with significant main effects or interactions (ES f), and for follow-up *post hoc t*-tests for dependent samples (ES dz) and for independent samples (ES d) using G^∗^power (Version 3.1.9.2). The statistical threshold of significance was set to α = 0.05.

### FMRI Activation

FEAT v6.00 was used to conduct hierarchical voxel-wise general linear model (GLM) analyses for pre-processed fMRI data. First-level modeling was conducted for each participant. Trials with block types (retrieval, motor, and probe) and rest modeled as separate regressors, convolved with a gamma hemodynamic response function, while rest blocks were treated as a baseline. Temporal derivatives were also included in the model. Contrasts of parameter estimates (COPEs) were calculated between retrieval and motor conditions, retrieval and rest, and motor and rest. The probe condition was not examined in the current analysis due to a lack of specific hypotheses regarding brain activation for this condition. A group level GLM for each COPE was conducted with FLAME (FMRIB’s Local Analysis of Mixed Effects) to assess the group average activation. In addition, separate two-group *t*-tests for each COPE were conducted to assess for sex differences. Four additional group-level GLM analyses were conducted to examine relationships between BOLD activation and performance measures; average total path length and latency to reach the platform in the retrieval condition were each examined as regressors in the higher-level model for the participant level retrieval COPE (retrieval versus rest), while these measures in the motor condition were examined as regressors in the higher-level model for the participant level motor COPE (motor versus rest). These variables were highly collinear in both retrieval and motor conditions, with a correlation of *r* = 0.779, *p* < 0.000 for path length and latency during retrieval and a correlation of *r* = 0.949, *p* < 0.000 for path length and latency during motor.

For all group level analyses, inference was done using Gaussian random field theory with cluster-based thresholding (*z* = 3.1) to control family-wise error, e.g., *p* < 0.05 corrected. Additionally, in order to understand specific increases or decreases relative to rest in frontal and hippocampal regions of interest during each condition, BOLD percent signal change was extracted from these regions for each contrast. Featquery was used with anatomical ROIs from the Harvard-Oxford Subcortical Structure Atlas to extract BOLD percent change for retrieval and motor conditions, from activated regions of the hippocampus, middle frontal gyrus (MFG), and anterior cingulate cortex (ACC) identified in the retrieval > motor contrast, and for activated regions of frontal medial cortex identified in the motor > retrieval contrast.

## Results

### Offline vMWT Performance

**FIGURE 4:**
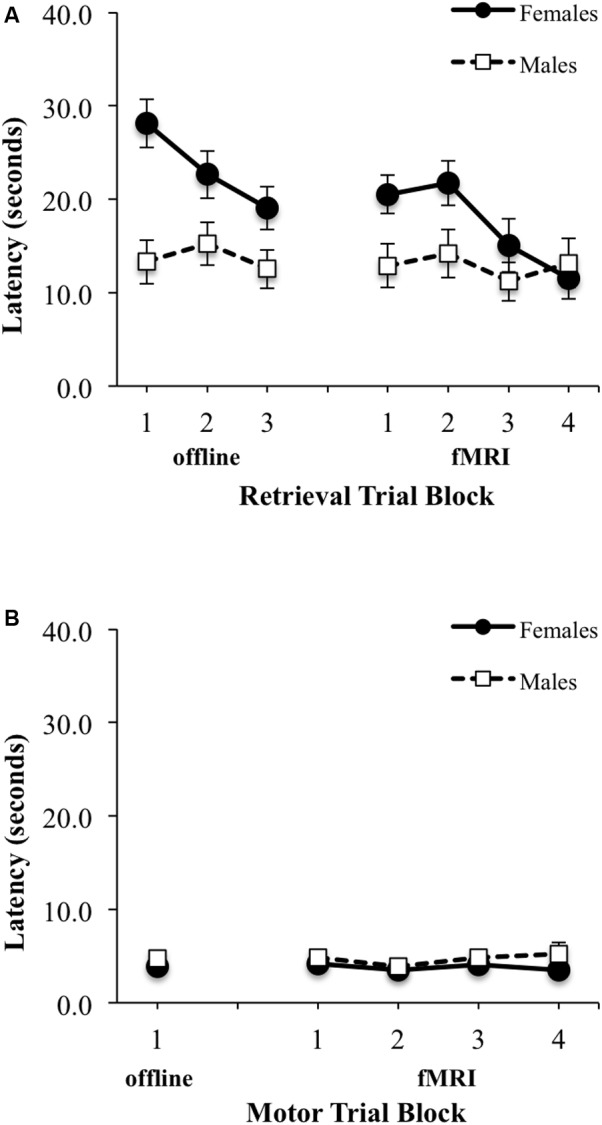
Latency (seconds) to reach the platform during: **(A)** three offline (left) and four fMRI (right) retrieval/hidden trial blocks; **(B)** one offline (left) and four fMRI motor/visible trial blocks. Females are represented as filled black circles with solid lines and males are represented as open squares with dotted lines.

**FIGURE 5:**
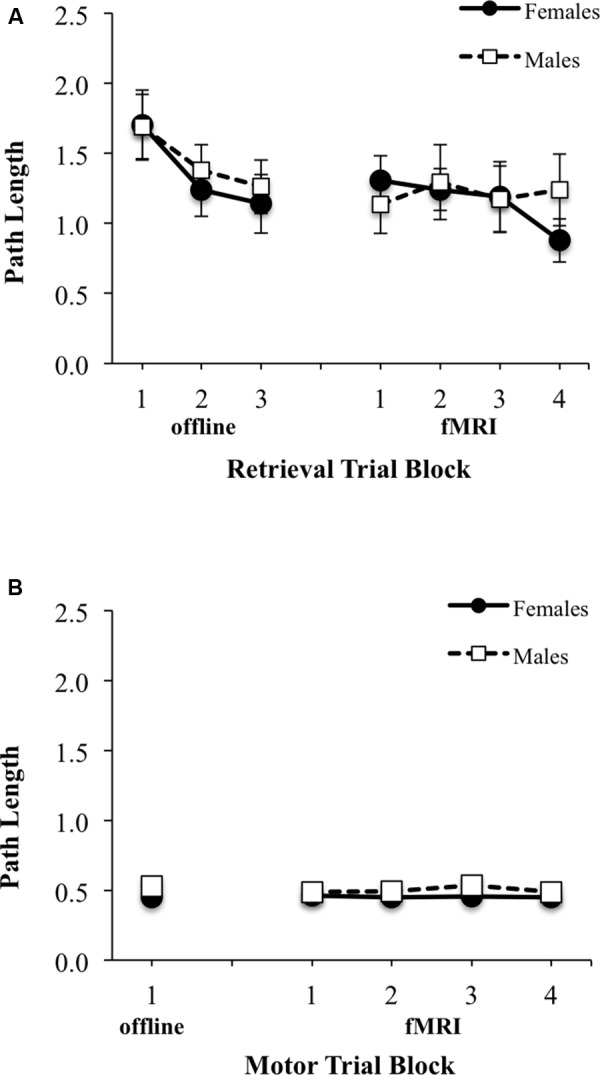
Path length (arbitrary units) from starting location relative to pool diameter during: **(A)** three offline (left) and four fMRI (right) retrieval/hidden trial blocks; **(B)** one offline (left) and four fMRI motor/visible trial blocks. Females are represented as filled black circles with solid lines and males are represented as open squares with dotted lines.

There was a significant main effect of block for latency to reach the platform during Learning/retrieval trials [*F*(2,58) = 9.24, *p* < 0.001, ES f = 0.57], with shorter swim latencies observed by blocks 2 and 3 relative to block 1 [block 1 vs. 2: *t*(30) = 2.34, *p* < 0.05, ES dx = 0.42; block 1 vs. 3: *t*(30) = 4.16, *p* < 0.001, ES dx = 0.74] (**Figure 4A**, top left). No significant sex by block interaction effect was observed, however, there was a main effect of sex for latency during learning/retrieval trials [*F*(1,29) = 8.72, *p* < 0.05; ES f = 0.55], with adolescent males demonstrating a shorter latency relative to adolescent females. Similarly, for path length, there was a significant main effect of block [*F*(2,58) = 6.03, *p* < 0.005, ES f = 0.46], with a shorter path length observed by blocks 2 and 3 relative to block 1 [block 1 vs. 2: *t*(30) = 2.96, *p* < 0.01, ES dx = 0.52; block 1 vs. 3: *t*(30) = 2.89, *p* < 0.001, ES dx = 0.51] (**Figure 5A**, top left). There were no significant interactions or main effects for sex or block. For the single block of motor trials, there were no significant sex differences for latency to reach the platform or path length (**Figures 4B**, **5B**, bottom left).

On the probe trial (fixed time length of 30 s), there were significant main effects of sex for latency [*F*(1,29) = 7.57, *p* < 0.05; ES f = 0.51], latency in region [*F*(1,29) = 10.74, *p* < 0.005; ES f = 0.51] and a trend for latency in quadrant [*F*(1,29) = 3.44, *p* = 0.07; ES f = 0.34] effect sizes. However, there were no significant sex differences for path length or percent total path length in the target region, path length or percent total in the platform quadrant, or heading error (**Table 2**). Percentage of overall path length, however, was significantly different than chance for all participants for both the region (5%) and the quadrant (25%) analyses (*p* = 0.000, for both one sample *t*-tests). No significant strategy preference was observed on the probe trial: 62.5% (*n* = 20) of adolescents utilized a direct strategy and 37.5% (*n* = 12) of adolescents utilized a non-direct strategy to reach the platform [χ^2^(1,32) = 2.00, *p* > 0.05]. Sex differences for strategy utilization did not reach statistical significance. Of the 62.5% using a direct strategy, 55% were female and 45% were male [χ^2^(1,20) = 0.20, *p* = 0.66], whereas for the 37.5% using a non-direct strategy, 33% were female, and 67% were male [χ^2^(1,12) = 1.33, *p* = 0.25].

**Table 2:** Offline probe performance data.

	Total sample (*N* = 31^a^)	Males (*N* = 17)	Females (*N* = 14)	*p*
First move latency	4.0 ± 1.7	3.3 ± 1.3	4.8 ± 1.8	0.01*
Latency to critical region	8.7 ± 7.2	5.3 ± 2.8	12.7 ± 8.8	0.003**
Path length to critical region	0.61 ± 0.32	0.57 ± 0.29	0.67 ± 0.36	0.39
% Path length in critical region	22.6 ± 15.0	21.6 ± 12.6	23.8 ± 17.8	0.69
Latency to quadrant	6.4 ± 7.1	4.3 ± 2.8	8.9 ± 9.8	0.07
Path length to quadrant	0.48 ± 0.31	0.46 ± 0.28	0.50 ± 0.35	0.71
% Path length in quadrant	52.9 ± 24.0	52.2 ± 25.1	53.6 ± 23.5	0.87
Heading error (degree)	22.1° ± 24.1	22.7° ± 26.1	21.3° ± 22.5	0.88


*Data represent mean ± standard deviation (*n* = 31). ^*a*^Offline data from one female participant was not available. There were no significant sex differences on any measure. ^∗^*p* < 0.05, ^∗∗^*p* < 0.005.*

### fMRI vMWT Performance

No significant sex by block interaction was observed, however, there was a significant main effect of block for latency to reach the platform during retrieval fMRI trials [*F*(3,90) = 3.16, *p* < 0.05; ES f = 0.32], with shorter swim latencies observed only between block 2 and block 4 [*t*(31) = 1.87, *p* < 0.05; EF dx = 0.41] (**Figure 4A**, top right). There also was a trend for a sex difference was observed for latency [*F*(1,30) = 4.15, *p* = 0.051, ES f = 0.37], with adolescent males demonstrating a shorter overall latency relative to adolescent females. For path length during fMRI, there were no significant interactions or main effects of block or sex (**Figure 5B**, top right). There were also no significant interactions or main effects of block or sex, for latency or path length for motor trials (**Figures 4B**, **5B**, bottom right). There were significant sex differences only in the average number of completed retrieval trials [*F*(1,30) = 6.11, *p* < 0.05; ES f = 0.45; males: 7.82 ± 4.19; females: 4.87 ± 2.10], but not in the motor trials [*F*(1,30) = 1.82, *p* = 0.19; ES f = 0.25; males: 16.88 ± 2.96; females: 15.80 ± 1.01].

### fMRI vMWT BOLD Activation

**FIGURE 6:**
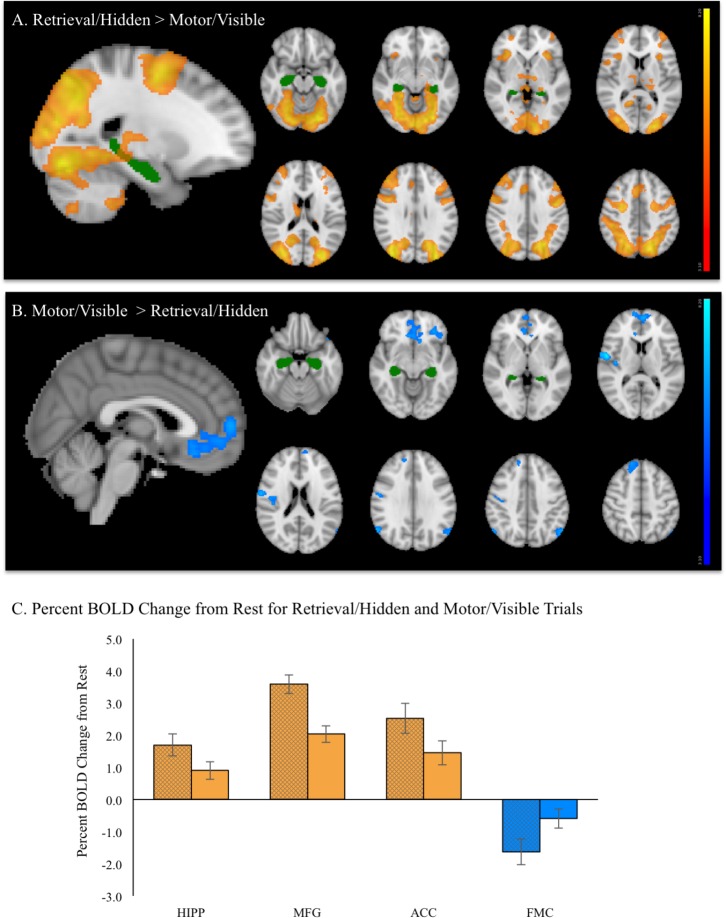
During fMRI vMWT performance: **(A)** Regions showing significant increases in brain activation (red–yellow, *p* < 0.05 corrected) during retrieval/hidden trials (with room cues) relative to motor/visible trials (with no room cues). **(B)** Regions showing significantly greater activation (blue–light blue, *p* < 0.05 corrected) during motor/visible trials relative to retrieval (hidden platform) trials. For reference, the hippocampus region of interest from the Harvard-Oxford Subcortical Structure Atlas threshold at 30% is shown in green. **(C)** Mean % BOLD signal change for retrieval/hidden > rest (solid bars with black cross hatch) and motor/visible > rest (solid bars) extracted from hippocampus (HIPP), middle frontal gyrus (MFG), anterior cingulate cortex (ACC), and frontal medial cortex (FMC). Orange indicates increased BOLD signal, blue indicates decreased BOLD signal.

A contrast of the retrieval (hidden) versus motor (visible) condition revealed five spatially extended clusters comprised of multiple brain regions. Regions within these clusters included portions of hippocampus and surrounding medial temporal lobe structures, such as parahippocampal gyrus. Significant activation was also observed in frontal cortex regions, including bilateral superior frontal gyrus (SFG), bilateral MFG, ACC and paracingulate gyrus and bilateral precentral gyrus. In addition to medial temporal and frontal areas, extensive activation was observed in visual processing areas such as bilateral fusiform gyrus and large bilateral areas of superior lateral occipital cortex. Activation also included the portions of posterior cingulate gyrus, thalamus, cerebellum, and brainstem (**Figure 6A**). A summary of the anatomical locations of local maxima for the clusters is provided in **Table 3**. In each region, mean activation was greater than rest in both retrieval/hidden and motor/visible blocks. This increased hippocampal activation relative to rest, however, as shown in the whole-brain analysis, was significantly greater for retrieval/hidden than motor/visible (**Figure 6C**, orange).

**Table 3:** Local maxima of activation: Retrieval/Hidden > Motor/Visible Contrast.

Region	Side	Volume (mm^3^)	z-max *df* = 31	MNI coordinates		
				
				*x*	*y*	*z*
*Extended region including:*		28304				
Fusiform gyrus	L		7.04	-24	-80	-14
Superior lateral occipital cortex	R		7.03	38	-70	24
	L		6.96	-32	-86	26
	L		6.85	-30	-82	24
	R		6.85	38	-86	30
	R		6.78	38	-82	28
*Extended region including:*		11659				
SFG	R		6.74	22	-2	58
SFG/MFG	R		6.72	26	-2	58
Precentral gyrus/MFG	L		6.41	-32	-8	56
SFG/MFG	L		6.29	-24	-2	58
Precentral gyrus	R		6.29	24	-8	48
SFG	L		6.23	-22	-6	54
*Extended region including:*		271				
Cerebellum	L		5.21	-30	-72	-56
	L		5.1	-30	-70	-50
	L		3.95	-18	-76	-50
*Extended region including:*		150				
Cerebellum	R		4.69	26	-72	-50
	R		4.16	36	-72	-52
	R		4.06	32	-70	-46
*Extended region including:*		146				
Brainstem			4.6	4	-32	0
Hippocampus/PCG	L		4.04	-4	-44	4
Thalamus	R		3.69	12	-32	-4


*Superior Frontal Gyrus (SFG); Middle Frontal Gyrus (MFG); Posterior Cingulate Gyrus (PCG).*

A contrast of motor > retrieval revealed seven clusters, many of which comprised multiple brain regions (**Figure 6B**). The largest of these clusters included bilateral frontal medial cortex, with activation extending into bilateral subcallosal cortex, frontal pole, ACC and paracingulate gyrus. Other frontal areas of activation included dorsal medial regions of right SFG and left frontal orbital cortex, with the latter cluster extending into left temporal pole. Significant activation also was observed in a right-lateralized cluster comprising pre- and postcentral gyrus, right insular cortex and right central opercular cortex. Other significantly activated regions for this contrast included portions of left and right superior lateral occipital cortex and right cerebellum, but not hippocampus. A summary of the anatomical locations of local maxima for the clusters is provided in **Table 4**. Notably, while retrieval > rest and motor > rest showed deactivation, the magnitude of deactivation was larger for retrieval than for motor (**Figure 6C**, blue), which contributed to the difference observed for the motor > retrieval contrast (**Figure 6B**).

**Table 4:** Local maxima of activation: Motor/Visible > Retrieval/Hidden Contrast.

Region	Side	Volume (mm^3^)	z-max *df* = 31	MNI coordinates		
				
				*x*	*y*	*z*
*Extended region including:*		1261				
Frontal pole, paracingulate gyrus	L		4.62	-4	58	6
Subcallosal cortex, FMC	L, R		4.45	0	30	-16
Frontal pole	R		4.2	12	64	8
Anterior subcallosal cortex	R		4.19	8	30	-10
ACC	R		4.15	10	34	4
Cingulate cortex/paracingulate gyrus	R		4.11	6	38	-2
*Extended region including:*		666				
Central opercular cortex	R		5.69	56	-2	10
	R		5.07	64	-2	6
Precentral gyrus	R		4.58	60	-2	16
Central opercular cortex/insular cortex	R		4.43	42	-14	14
Precentral gyrus	R		4.41	62	-4	20
Post-central gyrus/precentral gyrus	R		4.1	56	-8	28
*Extended region including:*		334				
Superior lateral occipital cortex	L		4.24	-56	-64	40
	L		4.09	-52	-66	42
	L		4.05	-50	-68	46
	L		4.03	-56	-66	34
	L		3.68	-60	-64	22
*Extended region including:*		286				
Frontal pole, SFG	R		4.3	16	42	44
Frontal pole	R		3.94	14	48	30
SFG, frontal pole	R		3.68	20	34	52
SFG	R		3.67	18	30	50
Frontal pole/SFG	R		3.48	8	44	48
*Extended region including:*		284				
Frontal orbital cortex	L		4.97	-44	28	-14
Frontal orbital cortex/frontal pole	L		4.02	-32	36	-10
Frontal pole	L		3.95	-36	40	-14
Temporal pole	L		3.77	-46	24	-22
*Extended region including:*		189				
Cerebellum	R		4.74	32	-82	-40
	R		3.61	46	-70	-38
*Extended region including:*		144				
Superior lateral occipital cortex	R		4.7	58	-64	34
	R		4.06	54	-66	42
	R		3.71	56	-60	46


*FMC, frontal medial cortex; ACC, anterior cingulate cortex; SFG, superior frontal gyrus.*

**FIGURE 7:**
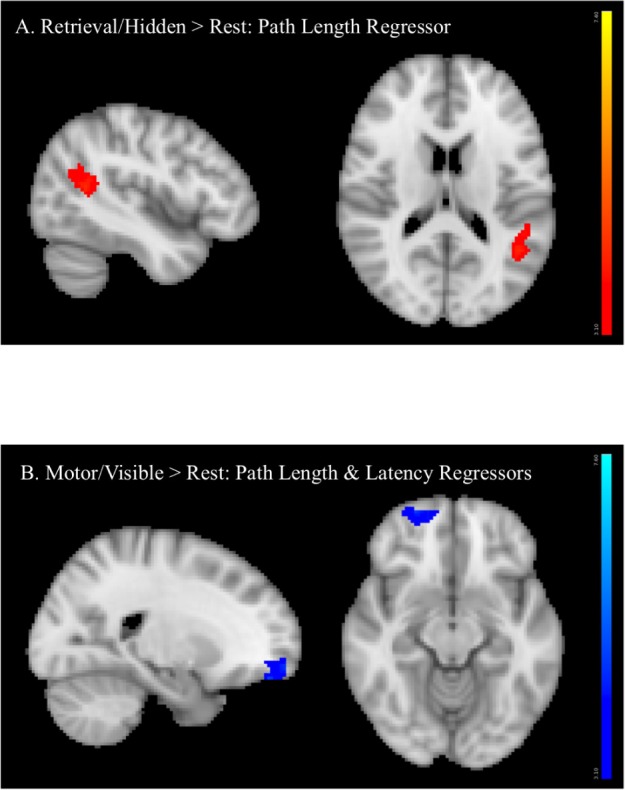
Regression analyses between BOLD activation and performance measures showing that **(A)** longer path length (orange) was associated with greater angular gyrus/supramarginal gyrus activation (retrieval > rest) and **(B)** longer latency (dark blue) and longer path length (lighter blue) (conducted in two separate analyses, activation superimposed in the same image) were associated with less frontal pole activation (motor > rest).

There were no significant sex differences for any COPE. Regression analyses conducted to examine relationships between BOLD activation and performance measures revealed that, in the retrieval condition, longer path lengths were significantly associated with greater activation of the angular gyrus/supramarginal gyrus (retrieval > rest, **Figure 7A**). In the motor condition, both longer latencies and path lengths were associated with less activation of the frontal pole (motor > rest, **Figure 7B**).

## Discussion

This study demonstrates fMRI evidence that a vMWT, modeled after the classic Morris task, elicits activation of the hippocampus and frontal lobe regions during memory retrieval in healthy adolescents. Importantly, this version of the vMWT reliably and rigorously measures spatial memory retrieval. Behavioral performance data confirm that participants could successfully perform and learn the task, based on decreased task latencies and increased number of trials completed during fMRI (reflecting speed of performance), and decreased path lengths to reach the platform location (indicative of better accuracy, independent of speed). Despite an extensive literature documenting robust sex differences in behavioral spatial memory performance (Astur et al., 2004; Andreano and Cahill, 2009; Voyer et al., 2017), only minimal sex differences were observed in the present study. In this healthy adolescent sample, males consistently demonstrated shorter latencies to reach the platform than females during both offline learning trials (Newhouse et al., 2007; Woolley et al., 2010; Sneider et al., 2015; Piber et al., 2018) and fMRI retrieval trials, which resulted in males also completing more retrieval/hidden fMRI trials. However, path lengths to reach the platform (offline learning and probe, and during fMRI) did not differ significantly between sexes, which is consistent with prior adolescent results (Sneider et al., 2015), but not with prior data reported from in prepubertal children (Newhouse et al., 2007) or in adults (Piber et al., 2018), in which males displayed significantly shorter path lengths than females when performing a vMWT. Nonetheless, these data provide subtle behavioral evidence consistent with a male advantage on the vMWT, in that females displayed the same accuracy as males (based on path lengths and probe performance), but at the expense of taking significantly longer to complete the trial. Stronger sex differences in retrieval may emerge beyond this particular age of adolescence (13–14 years old) with changes in pubertal status, as the proportion of males employing a more efficient strategy (direct spatial strategy) for reaching the platform location is greater than in females (Astur et al., 2002), and increases with age (Sneider et al., 2015).

Significant activation was observed in left hippocampus and posterior cingulate gyrus, as well as frontal executive regions that included bilateral SFG, bilateral MFG, and ACC. Importantly, hippocampal activation was observed during the retrieval > motor contrast, but not for motor > retrieval. Moreover, when retrieval and motor conditions were independently examined relative to rest, hippocampus was activated to a greater degree during retrieval than during motor. This pattern also was observed for frontal lobe regions including the MFG and ACC, again suggesting that these regions are active during processing of spatial information to a greater extent when memory retrieval is occurring as compared to motor performance alone. These findings are consistent with regional activation reported in middle-aged adults in our previous study that used a similar version of the vMWT, although acquired using a less efficient, non-multiband fMRI paradigm (Sneider et al., 2011). The lack of sex differences in brain activation is not consistent with adult findings; however, it is possible that either brain activation did not differ due to the similarities in path lengths (accuracy) on the task, or because the sample size was too small to detect sex differences in brain activation. Although to date there are no adolescent studies to compare the current results, overall, the regions of brain activation observed are similar to those observed during navigation of a familiar environment in adults, including bilateral hippocampus, posterior cingulate, MFG, and precuneus (Hirshhorn et al., 2012), and in adults during performance of a spatial discrimination learning task who exhibited significant hippocampal BOLD activation (Dahmani and Bohbot, 2015).

Analyses conducted to examine fMRI correlates of spatial memory performance showed that worse performance, measured as longer path lengths, was significantly associated with greater recruitment of the angular gyrus/supramarginal gyrus. In contrast, worse performance on motor trials, as evidenced as longer path lengths and longer latencies, was associated with reduced recruitment of the frontal pole. The regions of activation identified in these brain/performance associations in whole brain analyses were unique from regions activated in individual task contrasts (without performance regressors). Furthermore, no significant relationships were observed for performance measures and for brain activation from specific regions of interest (hippocampus, MFG, or ACC). Nonetheless, these brain-behavior relationships are not surprising, as the activated regions associated with performance measures have been implicated in spatial cognition and memory retrieval, including angular gyrus (Seghier, 2013), and for planning action sequences, mediated by the frontal pole (Okuda et al., 2003)). The angular gyrus, including the supramarginal gyrus, also plays a role in shifting of attention toward stimuli with high salience (toward task-relevant information, i.e., environmental cues in the vMWT) and particularly to retrieved memories (Ciaramelli et al., 2008). The angular gyrus also has been implicated in a multitude of functions involving distributed subsystems with brain regions that are involved in memory, attention, action and semantics (Seghier, 2013). Thus, greater activation of the angular gyrus might be present in worse navigators who make more errors, whereas less activation of the frontal pole might be present in worse navigators who are less effective in planning their navigation path. Notably, the current cohort consisted of healthy adolescents, examined within a narrow age range, who learned to perform the spatial and non-spatial tasks well and displayed little variability in task performance. These characteristics of the behavioral data could be one reason that specific regions of interest activated during retrieval (hippocampus, MFG, or ACC) were not significantly associated with performance.

A limitation of this study was the modest sample size of 32 adolescent participants, although this sample size is relatively typical for fMRI studies, and participants were healthy, well characterized and within a narrow age range. It is acknowledged that WASI IQ estimates were ∼1.5 standard deviations above the mean, which may not reflect the general population, and accordingly, could impact learning and memory measures, as well as BOLD signal. Preliminary sex differences were evident for some behavioral measures, but not for BOLD activation. A lack of BOLD differences could be due to a sample size that did not provide enough power to permit a full investigation of sex differences. Hence, sex differences reported, or lack thereof, should be interpreted with caution. A second study limitation was motion during the fMRI acquisition. However, participants with greater than 5 mm movement at any time point were excluded, after standard motion correction of remaining data, ICA-AROMA was applied to denoise motion effects from the fMRI data to further mitigate the impact of motion. Group level maps reflect clearly delineated brain regions that resemble well-known brain networks, thus the strategy used for motion reduction was successful. Finally, as the current vMWT paradigm acquired BOLD fMRI data during retrieval rather than during encoding (learning), it was necessary to establish that adolescent participants could successfully complete the task during fMRI (successfully retrieve the memory of the platform location), otherwise BOLD fMRI may have reflected navigation, rather than a longer-term memory process such as retrieval. Acquiring BOLD signal during learning could be accomplished, however, given the required efficiency of the block design and the relatively short, fixed length of hidden platform trials, it may have been more difficult for adolescents to learn the task for the first time while in the scanner. It also is plausible that participants continued to encode spatial information during fMRI, especially adolescent females who appeared to continue to show decreased latency over retrieval trials. These limitations should be addressed in future investigations.

## Conclusion

Data from this study demonstrate hippocampal activation when adolescents learned to use cues to navigate successfully in a virtual environment (retrieval memory). Activation during retrieval also was observed in several key frontal lobes critical for executive functioning, including planning, organizing, error monitoring, and decision-making. Thus, this translational task successfully targets neurocircuitry relevant for memory function in adolescents, which provides feasibility of this rigorous vMWT application for tracking developmental changes in integrated hippocampal and prefrontal neural activation and resource utilization (Murty et al., 2016). To this end, these adolescents are completing two additional annual neuroimaging assessments, along with assessments of perception of risk for alcohol and drug use, cognition, changes in mental health (e.g., depression and anxiety), and other behaviors particularly relevant to adolescent (e.g., risk taking, sensation seeking, impulsivity and delay discounting). As quarterly follow-up surveys and yearly visits reveal new alcohol and other drug use in particular, which is common during this passage of adolescence, the current data provide an important baseline for interpreting the impact of later initiation on the developmental maturation of hippocampal and frontal circuitry on the vMWT. Such examination of youth prior to behaviors that are maladaptive, e.g., alcohol and drug initiation, may also help establish a neurobiological signature that predicts who is at heightened risk for onset and continued use, and emerging issues with mental health (increased depressive and anxiety symptoms), which could concomitantly interfere with the incorporation of experience into adaptive behaviors required for transitioning through adolescence (Murty et al., 2016).

## Author Contributions

JS and MS were involved in all aspects of the research, including conception and design of the fMRI paradigm and study, analysis, interpretation, and writing the manuscript. DH was involved in task programming and data processing. JC-G was involved in the analysis, interpretation, and writing the manuscript. ES, NG, EO, and AS were involved in recruitment, study coordination, acquisition of all study data, and administration of the vMWT during fMRI. MR was involved in development of the multiband fMRI protocol on the 3T scanner. SH recruited participants and contributed to the concept and design of the study, interpretation of data, and review of the manuscript. LN provided training and oversight of all fMRI processing and data analysis. All authors read and approved the final manuscript.

## Conflict of Interest Statement

The authors declare that the research was conducted in the absence of any commercial or financial relationships that could be construed as a potential conflict of interest.

## Footnotes

1 https://github.com/rhr-pruim/ICA-AROMA
